# Scientometric trend analyses of publications on the history of psychology: Is psychology becoming an unhistorical science?

**DOI:** 10.1007/s11192-016-1834-4

**Published:** 2016-01-11

**Authors:** Günter Krampen

**Affiliations:** Leibniz Institute for Psychology Information (ZPID), 54286 Trier, Germany; Department of Psychology, University of Trier, 54286 Trier, Germany; Department of Psychology (INSIDE), University of Luxembourg, Luxembourg, Luxembourg

**Keywords:** History of psychology, Scientometry, Methodology, Publication genre, Psychology, Psychology education, Y800

## Abstract

Examines scientometrically the trends in and the recent situation of research on and the teaching of the history of psychology in the German-speaking countries and compares the findings with the situation in other countries (mainly the United States) by means of the psychology databases PSYNDEX and PsycINFO. Declines of publications on the history of psychology are described scientometrically for both research communities since the 1990s. Some impulses are suggested for the future of research on and the teaching of the history of psychology. These include (1) the necessity and significance of an intensified use of quantitative, unobtrusive scientometric methods in historiography in times of digital “big data”, (2) the necessity and possibilities to integrate qualitative and quantitative methodologies in historical research and teaching, (3) the reasonableness of interdisciplinary cooperation of specialist historians, scientometricians, and psychologists, (4) the meaningfulness and necessity to explore, investigate, and teach more intensively the past and the problem history of psychology as well as the understanding of the subject matter of psychology in its historical development in cultural contexts. The outlook on the future of such a more up-to-date research on and teaching of the history of psychology is—with some caution—positive.

## Introduction: sketch of the status of psychology in the sciences and its history

Historically, implementation of psychology as a discrete discipline within the canon of sciences is strongly related to the works of Wilhelm Wundt (1832–1920) in the German-speaking and most European countries and William James (1842–1910) in the Anglo-American countries. Sketchily, this “great men” approach to the history of psychology symbolizes very well its historical roots in the times before. In a less hagiographical, but rather a contextual and social history approach (see, e.g., Ball 2012) they symbolize as well the external surroundings and roots of the implementation of psychology. These roots and contextual factors refer to philosophy and the humanities (symbolized by: W. James originally was a philosopher, W. Wundt became one in his later years) as well as to the natural sciences (symbolized by: W. James became a psychologist using and propagating empirical methods, W. Wundt originally was a physiologist and physician and became a psychologist using and propagating experimental methods in his structuralism and hermeneutics in his precocious ethnic or cross-cultural psychology, i.e., the “Völkerpsychologie” as well; Wundt 1900–1929). Furthermore, these multidisciplinary roots of psychology in the nineteenth century are confirmed by the multidisciplinary scientific backgrounds and interests of the first psychology students at Leipzig, Harvard and Penn State University, which referred to the humanities (e.g., teachers and teacher students, philosophy and philology students, etc.) as well as to natural sciences (e.g., medical students and physicians, biology students, math students and mathematicians, etc.).

Thus, from its start not quite 150 years ago, psychology belongs in the canon of sciences to the humanities and to the natural sciences, and—furthermore—to the social sciences, too. This early relationship of psychological methodology to—both—quantitative experimental and qualitative hermeneutic methods has led time and time again to controversies and friction in psychology. Frequently ignored is the fact that both, in agreement, refer to empirical data (that, however, in different ways, i.e., nomothetic or more idiographic, respectively; see, e.g., Allport 1937; Bühler 1927; Stern 1900; more recently, see, e.g., Danziger 1995; Hurlburt and Knapp 2006; Windelband 1998). As a consequence, teaching about and research on the history of psychology is not only difficult (because it must consider both historical roots and both methodologies), it is also in danger of being caught between the stools of the various basic and applied subdisciplines of psychology with their differing methodological preferences and main streams. Between the stools can imply belonging to none or belonging to all subdisciplines, however, both cases with the inherent danger of being overseen, to debase its status, to disappear or—in the worst case—to be pulverized.

Such dangers were identified in early monographs on the “brief history of psychology” (e.g., Ebbinghaus 1908, p. 7; translation provided by the author) and—more prominently—in the “crisis of psychology” published in 1927 by Karl Bühler. Moreover, Bühler (1927) interpreted the methodological crisis of psychology as a transitional period of a young science (1900 a science, in which “psychologists were attempting to define themselves”; Tweney and Budzynski 2000, p. 1014), and he expounded as the solution that psychology requires experimental, hermeneutic, and behavioral methodologies and methods as well. His argumentation makes the crisis obsolete by the insight and knowledge that all “three psychological aspects” are a priori necessarily and adequate for the characterization of the subject matter of psychology.

Danziger (1994) refers—at least implicitly—to Bühler (1927) and broadens the argumentations in his question of whether “(…) the history of psychology (does) have a future” (Danziger 1994, p. 467). He ascertains and complains, that “the history of psychology tends to be accorded a purely pedagogical role within the discipline rather than being seen as a possible source of substantive contributions” and pulls this trend together with “a type of mobilization that is characteristic of the natural rather than the human sciences” (Danziger 1994, p. 467). These assertions are in line with Bühler (1927). However, he moves on to the distinction between a “shallow history of the scientific review” with the dominant pedagogical objective to “help to organize consensus” (and—this can be added—conformity in educational and research settings) versus the “critical history” representing “a threat to the moral community of researchers” (p. 467). While “shallow history” refers to regular, normal epochs of science of science that revolve round main stream research programs and paradigms including immunization strategies against falsifications, “critical history” has—at the very least—the potential for essential changes of research paradigms, i.e., that is, the potential for “scientific revolutions” (Kuhn 1970). Undoubtedly, scientific revolutions and significant changes in research paradigms are rather infrequent, but they are predicated on a critical, self-regulated learning and research that includes a critical history of the science under study. Danziger (1994) concludes with cautious optimism grounding in hopes—besides others—on the international diversification of psychology.

In the following, firstly, Danziger’s (1994) final argument is taken into consideration. Focus is on the trends and the recent situation of the research on and teaching of history of psychology in the German-speaking countries. Second, this is compared with the situation in other countries (mainly the United States, because this country dominates psychology internationally; see, e.g., Arnett 2008, 2009) by means of scientometric methods. Third, in accordance with others (see, e.g., Ball 2012; Danziger 1994, 1997; Pettit and Davidson 2014) attempts are undertaken to provide some impulses for the future of research on and teaching of history of psychology, including its status within the different subdisciplines of psychology. These impulses refer to (1) the necessity and significance of an intensified use of quantitative, unobtrusive scientometric methods in historiography in times of “big data”, (2) the necessity and possibilities to integrate qualitative and quantitative methodologies in historical research and teaching, (3) the reasonability of interdisciplinary cooperation of specialist historians, scientometricians, and psychologists, (4)—in accordance with Danziger (1994)—the meaningfulness and necessity to explore, investigate, and teach more intensively the past and the problem history of psychology as well as the understanding of the subject matter of psychology in its historical development in cultural contexts.

## Research on and teaching of history of psychology in the German-speaking countries

The German-speaking countries are Austria, Germany, Liechtenstein, and the German-speaking parts of Luxembourg and Switzerland. Frequently, these German-speaking nations are referred to as the DACHLL countries (D = Germany, A = Austria, CH = Switzerland, first L = Liechtenstein, second L = Luxembourg; note: in Switzerland and Luxembourg, German is one of each three different official languages with large dissemination and usage). Psychologists working in research and academic teaching are organized together in the transnational *Deutsche Gesellschaft für Psychologie* (DGPs; German Association of Psychology) founded in 1904. In contrast, applied working psychologists are organized in various national professional organizations because of the different professional, educational, health care, etc. laws.

### Research on the history of psychology in the German-speaking countries

Most psychologists who are engaged in research on the history of psychology in the German-speaking countries are organized in the section “History of Psychology” of the DGPs, which was founded in 1988. This is the smallest section of the DGPs with 59 members at present (the other sections have up to 640 members; *M* = 344, SD 193.6; Margraf 2015), undertaking small section conferences once every 2 years. Research is mainly individualized and somewhat like patchwork with identifiable foci on (1) biographical and autobiographical studies (i.e., a great men approach; see, e.g., Krampen 2009; Pongratz et al. 1979; Rattner 1995; Wehner 1992), (2) selected problems and theories of psychology (i.e., a problem history approach; see, e.g., Krampen and Montada 1998; Pongratz 1984), and (3) the ongoing attempts to analyze and cope with the history of German psychology in Nazi Germany, which started lately in the 1970s (i.e., a contextual, social, and professional history approach; see, e.g., Geuter 1984; Graumann 1985; Wolfradt et al. 2015). For a quantitative overview of the trends in research and publication activities on the history of psychology in the DACHLL countries, scientometric analyses were conducted by means of PSYNDEX, the database for German- and English-language psychology publications from the Germany-speaking countries.

### Scientometric methods

All data used in the following derive from PSYNDEX, which is produced by the Leibniz Institute for Psychology Information (ZPID; Trier, Germany). PSYNDEX is the database for German- and English-language publications in psychology and its neighboring disciplines in the German-speaking countries (i.e., DACHLL). Documentation starts exhaustively with the publication year 1980 (for German psychological tests: 1945), before this documentation is selective. At the beginning of 2015 there are about 300,000 documents in PSYNDEX (retrieval, e.g., from www.zpid.de, www.MEDPILOT.de, or www.pubpsych.de). From the basic population of the database, samples of publications were selected by means of search strategies that refer to publications on the main topic “history of psychology” (date of searches: February, 2015). Scientometric analyses refer to the documentation and search fields (see *APA Thesaurus of Psychological Index Terms*, Gallagher Tuleya 2007; *PSYNDEX Terms*, ZPID 2011) “Classification Code” (CC), “Year of Publication” (YP), “Publication Type” (PT), “Subject Headings” (SH), and “Keywords” (MP).

### Results

#### Publications on the history of psychology in DACHLL countries 1980–2014

**Fig. 1:**
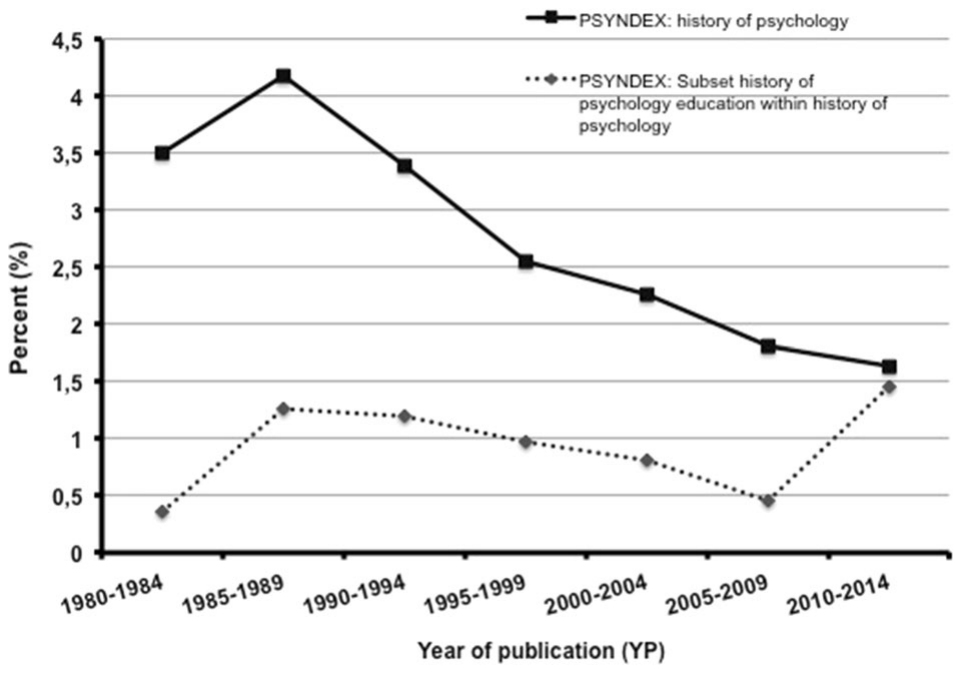
Relative frequencies (%) of publications on the history of psychology with reference to all publications and of publications of the subset “history of psychology education” with reference to publications on the history of psychology in PSYNDEX

In total, there are 8130 publications with the main topic history of psychology documented in PSYNDEX, which is 3.07 % of all psychological publications documented from the DACHLL countries (see right columns in Table 1). For 5-year-intervals (referred to in the following as “quintades” in analogy to decades) there is a marked drop of absolute and relative publications frequencies since the 1990s with a maximum of 4.2 % in the quintade 1985–1989 and the minimum of 1.6 % in 2010–2014 (see Fig. 1). Absolute frequency of publications on the history of psychology from DACHLL countries is statistically not significantly correlated with the number of all psychological publications from DACHLL, which show an increase in the time under study (*r* = .24; *p* > .10).

**Table 1:** Absolute and relative frequencies of publications on the history of psychology with reference to all publications documented as well as that of the subset of publications with the main topic “psychology education” in PsycINFO and PSYNDEX (psychology database from the German-speaking countries)

Database	PsycINFO		PSYNDEX	
(SH) Subject heading^a^ (CC) Classification code^a^	*f*	%	*f*	%
*(SH) History of psychology (Σ)*	28,511	0.73	8130	3.07
In publication years (YP)				
YP = 1804–1979	6611	0.82	397	2.88
YP = 1980–1984	1910	1.01	832	3.50
YP = 1985–1989	2907	1.09	1579	4.18
YP = 1990–1994	2823	0.92	1499	3.39
YP = 1995–1999	3027	0.94	1137	2.56
YP = 2000–2004	4066	0.96	985	2.26
YP = 2005–2009	3703	0.54	873	1.82
YP = 2010–2014	3464	0.40	828	1.64
*(SH) Psychology education and (SH) History of psychology (Σ)*	471	1.65	80	0.98
In publication years (YP)				
YP = 1804–1979	103	1.56	4	1.01
YP = 1980–1984	30	1.57	3	0.36
YP = 1985–1989	64	2.20	20	1.27
YP = 1990–1994	58	2.05	18	1.20
YP = 1995–1999	42	1.39	11	0.97
YP = 2000–2004	93	2.29	8	0.81
YP = 2005–2009	48	1.23	4	0.46
YP = 2010–2014	33	0.95	12	1.45

*f* frequency, % percent, *CC* classification code, *SH* subject heading, *YP* year of publication

^a^Thesaurus of Psychological Index Terms (Gallagher Tuleya 2007; ZPID 2011)

#### Historical publications within different subdisciplines of psychology in DACHLL

The total sample of 8130 PSYNDEX documents on the history of psychology is differentiated by the logical operation “and” for the different classification codes (CC) systemizing subdisciplines of psychology in the *APA Thesaurus of Psychological Index Terms* (Gallagher Tuleya 2007; ZPID 2011). Most publications (47 %; see Table 2) are classified in CC “General: History & Systems of Psychology”, which is—at first glance—rather trivial.

**Table 2:** Absolute and relative frequencies of publications on the history of psychology with reference to all publications documented for different psychological subdisciplines (Classification Codes, CC) in PsycINFO and PSYNDEX (psychology database from the German-speaking countries)

Database	PsycINFO		PSYNDEX	
(SH) Subject heading^a^ (CC) Classification code^a^	*f*	%	*f*	%
*(SH) History of psychology (Σ)*	28,511		8130	
In classification code (CC)^b^				
(21*) General: history and systems	8920	14.5	3983	46.8
(22*) Psychometrics, statistics and methodology	618	0.3	276	0.8
(23*) Human experimental psychology	1929	0.7	641	2.0
(24*) Animal experimental and comparative psychology	300	0.3	29	2.2
(25*) Physiological psychology and neuroscience	617	0.2	122	0.7
(26*) Psychology and the humanities	1616	0.5	692	11.2
(27*) Communication systems	254	0.4	97	0.9
(28*) Developmental psychology	907	0.4	385	1.6
(29*) Social processes and issues	1371	0.6	569	1.4
(30*) Social psychology	732	0.6	189	1.6
(31*) Personality psychology	4543	3.2	1916	10.1
[Sub CC (3143) Psychoanalytic theory	3240	71.3^c^	1708	89.1^c^]
(32*) Psychological and physical disorders	1709	0.2	552	0.7
(33*) Health and mental health treatment and prevention	5163	0.7	2203	2.4
[Sub CC (3315) Psychoanalytic therapy	1499	29.0^c^	1129	51.2^c^]
(34*) Professional psychological and health personnel issues	1631	1.2	471	3.3
(35*) Educational psychology	862	0.2	303	1.1
(36*) Industrial and organizational psychology	404	0.2	133	0.6
(37*) Sport psychology and leisure	80	0.3	30	0.7
(38*) Military psychology	89	0.5	27	6.1
(39*) Consumer psychology	52	0.1	13	0.3
(40*) Engineering and environmental psychology	83	0.2	67	0.9
(41*) Intelligent systems	74	0.2	8	0.9
(42*) Forensic psychology and legal issues	89	0.2	30	0.5

*f* frequency, % percent, *CC* classification code, *SH* subject heading, *YP* year of publication

^a^ *Thesaurus of Psychological Index Terms* (Gallagher Tuleya 2007; ZPID 2011)

^b^Including the possibility of double classifications (CC)

^c^Percentage with reference to frequency of CC = 31* or CC = 33*, respectively

Other, somewhat higher, percentages of publications on the history of psychology refer to CC “Psychology & the Humanities” (11 %) and CC “Personality Psychology” (10 %). The last result is due to the very high number of publications on the history of psychoanalytic personality theory (see Table 2). There is a similar result for the subclassification CC “Psychoanalytic Therapy”, which constitutes more than the half of all historical contributions to CC “Health & Mental Health Treatment & Prevention” (see Table 2). Furthermore, visible, but low percentages of historical studies are present in CCs “Military Psychology” (6 %; but, be aware of the very low absolute number of publications, which refer mainly to the history of Nazi military psychology), “Professional Psychological & Health Personnel Issues” (3 %), “Animal Psychology” (2 %; but, again, a low absolute number), and “Human Experimental Psychology” (2 %). The percentage of historical contributions to all other psychological subdisciplines is lower than 2 %, down to 0.5 % (see Table 2). These are percentages, which suggest these subdisciplines to be rather ahistorical ones.

#### Publication types in the literature on the history of psychology in DACHLL

The total number of publications on the history of psychology from the DACHLL countries is, furthermore, analyzed for the frequency of different publication types. Results presented in Table 3 show that publications on the history of psychology is loosing its character as a “book science” and is becoming a “journal science” since the 1970s. This trend follows the even more pronounced similar trend in psychological publications in total (see, e.g., Schui et al. 2014). The increase in journal publications on the history of psychology is accompanied by more publications in the form of book chapters and edited books. The increasing frequencies of reprints and dissertations suggest optimism. However, it should be considered that the first result may be an artifact that is possibly pushed by books on demand, which are documented in databases, but rather seldom bought and read, and that the second result is not in accordance with the much higher increase of dissertations in psychology overall (see, e.g., Schui et al. 2014).

**Table 3:** Absolute and relative frequencies of different publication types with reference to all publications on the history of psychology in PsycINFO and PSYNDEX (psychology database from the German-speaking countries)

Database	PsycINFO		PSYNDEX	
(SH) Subject heading^a^ (PT) Publication type^a^	*f*	%^b^	*f*	%^b^
*(SH) History of psychology (Σ)*	28,511	100	8130	100
Publication type (PT)				
PT = Journal article	18,741	65.7	3976	48.9
PT = Book chapter and edited book^c^	5556	19.5	1980	24.4
PT = Authored book	2611	9.2	1305	16.1
PT = Reprint	298	1.0	118	1.5
PT = Dissertation (abstract)^d^	1281	4.5	348	4.3
PT = other (e.g., AV media)	0	0.0	403	5.0
PT = Journal article				
YP = 1804–1954	922	5	12	0.3
YP = 1955–1974	1789	10	32	1
YP = 1975–1994	6416	34	2174	55
YP = 1995–2014	9614	51	1758	44
PT = Book chapter and edited book^c^				
YP = 1804–1954	1423	26	3	0.2
YP = 1955–1974	311	6	4	0.2
YP = 1975–1994	1037	19	902	46
YP = 1995–2014	2785	50	1071	55
PT = Authored book				
YP = 1804–1954	1319	51	6	0.5
YP = 1955–1974	220	8	18	1
YP = 1975–1994	426	16	614	47
YP = 1995–2014	646	25	667	51
PT = Reprint				
YP = 1804–1954	0	0	3	3
YP = 1955–1974	4	1	16	14
YP = 1975–1994	141	47	14	12
YP = 1995–2014	144	48	85	72
PT = Dissertation (abstracts)^d^				
YP = 1804–1954	3	0.2	27	8
YP = 1955–1974	60	5	73	21
YP = 1975–1994	602	47	202	58
YP = 1995–2014	616	48	101	29

*f* frequency, % percent, *CC* classification code, *SH* subject heading, *PT* publication type, *YP* year of publication

^a^ *Thesaurus of Psychological Index Terms* (Gallagher Tuleya 2007; ZPID 2011)

^b^Rounded to nearest whole numbers

^c^Not discriminable because documentation of book chapters includes the edited book

^d^Dissertation abstracts in PsycINFO, documentation of dissertations in PSYNDEX

### Teaching of the history of psychology in the German-speaking countries

#### Developments over the last decades

Since the 1990s there has been a continuous drop in the teaching of the history of psychology in undergraduate and graduate psychology education curricula in the DACHLL countries. This trend was markedly pushed on by the transformation of the European Diploma psychology study programs to Bachelor (BSc) and Master of Science (MSc) psychology study programs after the millennium.

Before this transformation, undergraduate education (“Vordiplom”) focused on psychological methods and the basic subdisciplines of psychology, while postgraduate education (“Hauptdiplom”) focused on sophisticated research methods and the applied subdisciplines. Governmental laws and specifications require in Europe for BSc studies both aspects to be covered, that is, teaching the basic and the applied subdisciplines (at a basic level) as well as methodology, and MSc studies must focus on elaborated and intensive education and training in selected subdisciplines with an applied and/or a research focus. Thus, teaching of the history of psychology was dropped in many departments of psychology, because a large number of psychological subdisciplines must be packed in the BSc curricula thus leaving little room or time for teaching the history and systematics of psychology. In addition, most faculties of psychology had and have no specialized department for the history (and systematics) of psychology, at best we find a psychology department with a combined focus at some universities (e.g., Adolf-Würth Institute for the History of Psychology at the University of Würzburg; Department of General and Theoretical Psychology at the University of Heidelberg; Department of Clinical Psychology, Psychotherapy, and Science Research at the University of Trier).

#### Recent situation of history of psychology teaching in the DACHLL countries

At present, the history of psychology is part of the BSc psychology curriculum in less than 10 of approximately 90 universities in the German-speaking countries with basic and major psychology education (Abele-Brehm et al. 2014). The history of psychology is represented as a subject in none of the MSc psychology curriculums (Allesch et al. 2015; Abele-Brehm et al. 2015). However, many colleagues argue that they integrate the special history of their subdiscipline in their lectures, seminars, courses, and exams. This may be better than nothing, but in reality this strategy neither assures the teaching of the systematics and general, integrative history of psychology with reference to its historical contexts nor the teaching of the history research methodology in a motivating setting. Personal, therefore selective, impressions of such attempts to teach the history of this specific subdiscipline as somewhat as an advanced organizer refer in many cases more to presenting a hit parade of highly selected historical experiments and/or theories, which sometimes resemble more a quasi-homage to a top of the flops (with an attempt to provide entertaining excerpts to students’ to elicit their amusement and laughter) than to a serious, but perhaps also student-motivating instruction.

#### Publications on the history of psychology for use in psychology education in PSYNDEX

Only 80 of the total of 8130 publications on the history of psychology from the DACHLL countries refer to psychology education (0.98 %; see Table 1). Because of the small base rate of these publications, a clear developmental trend is not visible. Figure 1 suggests a continuous decrease since the late 1980s and an unexpected, sudden increase from 0.5 % in the quintade 2005–2009 up to 1.5 % in 2010–2015. De facto this finding is the result of two editions deriving from anniversary symposia commemorating the Departments of Psychology of two German universities, in which the alumni reminisced and reflected on their study experiences years ago. Thus, an increase in the number of publications on the history of psychology with an educational and/or teaching objective in the most recent quintade under study is delusive.

## Comparison of trends concerning publications on the history of psychology in the German-peaking versus other countries (mainly the US)

In the following, some of the scientometrically obtained historiographical results on trends and the recent situation of publications on the history of psychology for the German-speaking countries presented in the paragraph above are briefly compared with the trends and the situation in other countries (mainly the US, because the US dominates psychology internationally). Specifically, scientometric methods will be applied to the PsycINFO database.

### Scientometric methods

All data used in the following derive from PsycINFO, which is produced by the *American Psychological Association* (APA). The APA highlights PsycINFO to be an international database going back to 1806. However, PsycINFO is dominated markedly by Anglo-American and English-language publications (>90 % of the documents (publications from the US: approximately 65 %). Its coverage of Anglo-American psychological publications improves, becoming very good, but not before the late 1970s in the context of digitalization. Less than 2 % of PsycINFO documents refer to English- and German-language publications from the German-speaking countries. Only 512 of the 8130 English- and German-language publications on the history of psychology from the DACHLL countries in PSYNDEX (see Table 1) are documented in PsycINFO. Thus, the coverage, at 6.3 %, is very low. At the beginning of 2015 PsycINFO contains approximately four million documents (retrieval, e.g., from http://www.apa.org/pubs/databases/psycinfo/index.aspx). Thus, we must keep in mind that PsycINFO contains more than 13.3 times more documents than PSYNDEX and—therefore—all comparisons between the frequencies found in PsycINFO and PSYNDEX must be implemented by means of within-database relativization in terms of percentage.

### Results

#### Publications on the history of psychology in PsycINFO

**Fig. 2:**
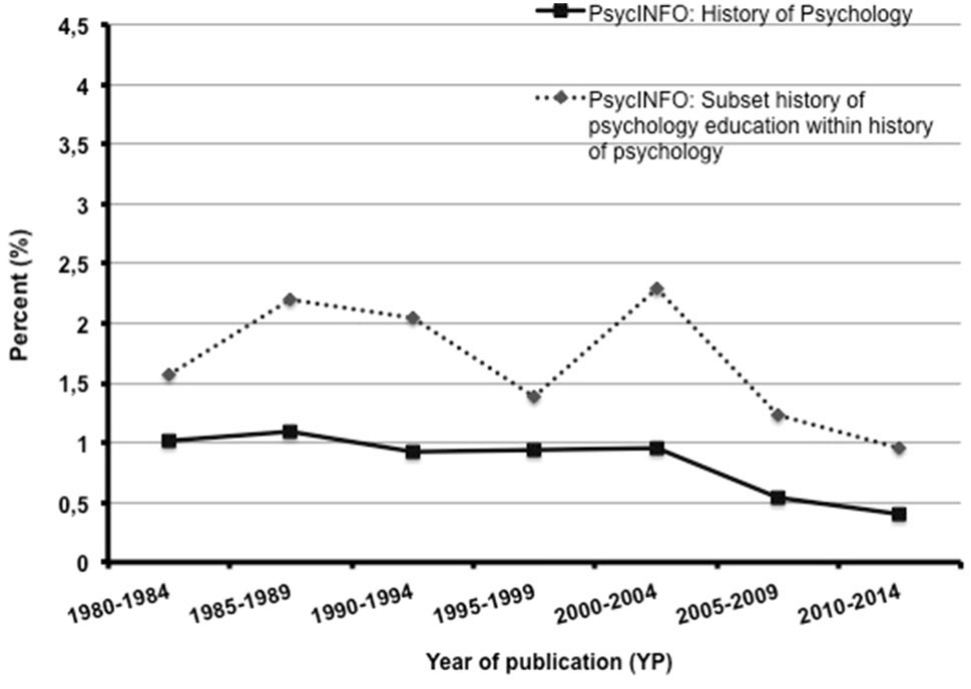
Relative frequencies (%) of publications on the history of psychology with reference to all publications and of publications of the subset “history of psychology education” with reference to publications on the history of psychology in PsycINFO

In total, there are 28,511 publications on the history of psychology documented in PsycINFO. This is 0.73 % of all the documented psychological publications (see left columns in Table 1) and, in relative terms, this value is markedly less than in PSYNDEX (3.07 %). In accordance to PSYNDEX, there is a drop of absolute and relative publications frequencies, but—in comparison—the drop has a time delay of a decade and happened after the millennium with a maximum of 1.09 % in the quintade 1985–1989 and the minimum of 0.40 % in 2010–2014 (see Fig. 2). The slight maxima shortly before and after the centennial of the *American Psychological Association* (APA) in 1992 may be interpreted as a history effect, however, if so, this history effect is very small (see Fig. 2). Absolute frequency of documented publications on the history of psychology in PsycINFO is statistically not significantly (but in tendency negatively) correlated with the number of all psychological publications, which show a very strong increase in the time under study (*r* = −.19; *p* > .10).

#### Historical publications within different subdisciplines of psychology in PsycINFO

The total sample of PsycINFO documents on the history of psychology is differentiated by the logical operation “and” for the different classification codes (CC) systemizing subdisciplines of psychology delineated in the *Thesaurus of Psychological Index Terms* (Gallagher Tuleya 2007; ZPID 2011). In PsycINFO, only 14.5 % of the publications on the history of psychology are classified to CC “General: History & Systems of Psychology”, which is much less than in PSYNDEX (47 %; see Table 2) and, therefore, the “first impression” that the PSYNDEX result is rather trivial must be revised. However, there are rather few subdisciplines of psychology with somewhat visible percentages of publications on the history of the particular subdiscipline. With two exceptions all percentages are lower than 1 %. We must bear in mind the much higher base rates of publications from all subdisciplines in PsycINFO, which reduce the percentages of special topics and themes. The two exceptions refer, in accordance with the PSYNDEX results, to CC “Personality Psychology” (again with very high portion of the subclassification CC “Psychoanalytic Personality Theory”) and to CC “Educational Psychology” (see Table 2). To sum up, in accordance with PSYNDEX, the results obtained from PsycINFO affirm the hypothesis that publications about and from most subdisciplines of psychology only very seldom feature their history as a main topic.

#### Publication types in the literature on the history of psychology in PsycINFO

Results on the relative frequencies of different publication types in the literature on the history of psychology documented in PsycINFO are in line with the PSYNDEX results presented above (see Table 3). Both scientometric, historiographical analyses show that, since the 1970s, literature on the history of psychology is losing its character as a “book science” and is becoming a “journal science”. This accords with a general, even more pronounced trend of psychological publication types overall (see, e.g., Schui et al. 2014) and is in line with Danziger’s (1994, p. 467) argument on parts of modern psychology “reflects a type of mobilization of tradition that is characteristic of the natural than the human sciences”. Natural sciences are the forerunners and pushers of (short, but many) journal publications, while the humanities and social sciences historically have been the classical “book sciences.” This arrangement has been changing in the last decades, and can be recognized by the decrease in authored books and by the increase in edited books and book chapters as well as in journal publications.

#### Publications on the history of psychology for use in psychology education in PsycINFO

At least 471 out of 28,511 publications on the history of psychology documented in PsycINFO refer to psychology education (1.65 %; see Table 1), which, in relative terms, is somewhat more than in PSYNDEX (0.98 %). As is the case for PSYNDEX, because of the small base rate of these publications, a clear developmental trend is not visible for PsycINFO as well. Figure 2 suggest an up and down movement, in which the peaks are—at least in part—a result of the few specialized edited textbooks on the history of psychology predominantly published with reference to graduate education (*n* = 129 documents) rather than to undergraduate education (*n* = 29 documents).

## Discussion and outlook on the future and some impulses for research on and teaching of the history of psychology

Before closing, in accordance with others (see, e.g., Ball 2012; Danziger 1994; Pettit and Davidson 2014) and continuingly it will be attempted to formulate some impulses for the future of an up-to-date, both student’ and colleague’ motivating research on and teaching of the history of psychology. This attempt includes the promotion of its status within the different subdisciplines of psychology to stimulate interest and engagement in as well as research on and the curricular defined teaching of the history of psychology. First, I stress the necessity and significance of an intensified use of quantitative, unobtrusive scientometric methods in historiography in times of “big data.” Second, I argue for the necessity and possibilities to integrate qualitative and quantitative methodologies in historical research and teaching. Third, I provide support for the reasonability of interdisciplinary cooperation of specialist historians and psychologists, at least in specific research projects. Fourth, I underline the meaningfulness and necessity to explore, investigate, and teach more intensively the past and the problem history of psychology as well as the understanding of the subject matter of psychology in its historical development in cultural contexts. Some of these impulses may be fruitful for some other sciences, social sciences and humanities as well.

### More use and appreciation of quantitative scientometrics in historiography

Research on and teaching of the history of psychology must more intensively extend the leading methods of the person (“great men”) approach, problem history approach, history of thought approach (with its concept of evolvement), and social history approach to quantitative scientometric methods, semantic technologies, and time series analyses in historiography (see, e.g., Green et al. 2013, 2014, 2015; Simonton 2006, 2014). Up to now, scientometrics and time series analyses are frequently criticized and devaluated in historical research and instruction because of their alleged purely descriptive, superficial, and cursory approach. Quantitative methods—frequently added on somewhat arrogant—do not promote understanding and cannot result in substantive contributions.

First of all, all these devaluations are assumptions, which obviously overshoot the *objectives of scientometrics* and time series analyses referring *de facto* to descriptive and comparative historiography—so far, so good. However beyond that, especially comparative historiography can promote and provide some insights and understandings in the history and current state of research on and teaching of history of psychology as well. The two sections before may show this with reference to the diversification (or lack of diversification, respectively) of psychology in different national and multinational research communities, which Danziger (1994) argued for more than 20 years ago rather intuitively and based primarily on impressions.

Second, all historical approaches and methods have their own advantages, but disadvantages, too: The person (“great men”) approach tends to ignore contextual historical factors and to overestimate individual geniality (Ball 2012); the problem history approach is frequently very selective and tends to stabilize the main stream in review form; the history of thought approach, with the dominant concept of evolvement and the pseudoargument of *Zeitgeist,* suffers from its necessary and unavoidable selectivity and—therefore—subjectivity (an optimization of this is, of course, the cognitive-historical approach; Tweney 2013); and the social history approach, with its focus on contextual factors, is frequently overburdened by the multidimensionality, multivariate, and interactive character of the contextual factors, which easily results in very reduced, therefore selective and—thus—subjective descriptions and interpretations. In this canon of historical methods, the advantages of quantitative scientometric methods and time series analyses in historiography lie in the fact that they are *unobtrusive methods* in terms of Webb et al. (1966), because they use artifacts (i.e., documentation) of psychological publications in databases with general access. Thus, this is a very good complement to other historical research methods.

A third argument in favor of scientometrics and time series analyses in research on and the teaching of the history of psychology refers to the frequently mentioned *information overload* in modern times, which simply “exploded” with digitalization and the Internet and is sometimes referred to as information explosion or—rather optimistically—knowledge explosion. First in the line of recent developments are scientific information and publications. It is literally impossible for an individual to handle, read, absorb, understand, and review the huge bulks of information: “Big data” require other analytical methods, for example, scientometrics (e.g., Krampen and Montada 1998) or “histiometrics” (Simonton 2006, 2014), time series analyses (e.g., Krampen et al. 2011), semantic technologies and text mining (see, e.g., Green et al. 2013, 2014, 2015), and visual displays of information (see, e.g., Smith et al. 2002).

This third argument implies yet another, more *pragmatic advantage of using scientometrics* more intensively in research on and the teaching of history of psychology: Its application and implementation converges with the digital world, a place where both students and colleagues alike are living in. Thus, the method is motivating and mirrors the challenges faced in everyday life. Above all, scientometric results are frequently presented in graphs, which “provide a compact, rhetorically powerful way of representing research findings” (Smith et al. 2002, p. 749) in journals and in teaching. Combined with some sort of “experiential history” (Boynton and Smith 2006) simulating and—more important—replicating significant historical experiments and empirical studies (Open Science Collaboration 2015) may be a good way to reduce the disinterest of to many students and colleagues towards the history of psychology.

Last but not least, it should be mentioned that the frequently mentioned criticism that scientometrics are conservatively stabilizing mainstream research is a misconception. Surely, scientometrics are implemented to describe and compare historical trends and foci of research. But this is not the whole issue. Scientometrics provide wonderful, effective, and efficient auxiliary means for the *identification of research deficits and gaps*, especially in comparative historiography referring to different research communities and/or epochs. This can be implemented freehand, but functions better by means of ontologies of scientific terms (e.g., the *Thesaurus of Psychological Index Terms*, Gallagher Tuleya 2007; *PSYNDEX Terms*, ZPID 2011) or by careful reconstructions of the terminology and language of a science (for psychology, see, e.g. Danziger 1997; Pongratz 1984).

### Integration of qualitative (hermeneutic) and quantitative (scientometric) methodology in historiography

At least since Bühler (1927), the necessity of an integration of qualitative and quantitative methods in psychology is a truism, which is—however—frequently forgotten or completely ignored. This truism follows not only from the insight that the subject matter of psychology is defined a priori by “three psychological aspects” (Bühler 1927, p. 29) requiring experimental, hermeneutic, and behavioral research methods, but also from the more recent principle of multiple measurement to assure the objectivity, reliability, and validity of data and of research results. However, an integration of research methodologies is missing up to now, perhaps because mainstream psychology and—even more—more or less self-contained specialist and/or national research communities tend to protect their methodological preferences *inter alia* by means of ignorance and devaluation of other(s’) methods.

Elsewhere, and in other psychological research contexts (i.e., research on creativity and divergent thinking, on psychotherapeutic practice expertise and evidence-based psychotherapy, as well as on science and scientists’ evaluations by means of peer reviews or expert evaluations, respectively, and psychometrics, randomized controlled trials, or scientometrics, respectively), a *pragmatic attempt for the integration of quantitative and qualitative methods* was undertaken (Krampen 2008, 2010, 2012, 2013). On the *first level,* quantitative methods (e.g., psychometric tests on divergent action and thinking, randomized controlled psychotherapy efficacy studies or scientometrics) are implemented to measure and confirm empirically the more general requirements of, for example, experts’ social evaluations of creativity, psychotherapists’ decisions on different indications, or scientific productivity, respectively, with the utmost objectivity, reliability, and validity as possible. This includes the possibility of direct inter- and intraindividual comparisons of individuals or groups and complies with the nomothetic model and philosophy of sciences. Located on the *second level* are the experts’ social evaluations, specialist psychotherapists’ (or expert physicians’) treatment decisions, or peer reviews of scientific papers and research proposals, respectively. This is more individualized and integrative oriented considering not only the empirical quantitative scientific state of the art, but all other significant information and the expertise of the experts, psychotherapists, and peer reviewers, respectively, as well in a more idiographic and problem-centered approach, which complies with the phenomenological, hermeneutic model and philosophy of sciences. Neither the first nor the second level is better than the other or more or less scientific, because (1) level 1 is a priori a necessary, but not sufficient premise for level 2 and (2) level 2 must include the information of level 1—if this is not the case, the evaluations, interpretations, decisions, or reviews at level 2 are not empirically based and are—therefore—in danger of being subjective, perhaps even arbitrary and ultimately wide open to criticism and devaluations.

This approximate description of the *integrative pyramid model for quantitative and qualitative research methods* is open for more levels in between the two briefly described main levels. Up to now, this has been exemplarily elaborated for research and practice with reference to creativity and divergent thinking (see Krampen 2013) and psychotherapy (Krampen 2010). The time is ripe for an exploration of the model in the context of research on the history of psychology.

### Interdisciplinary cooperation of specialist historians, scientometricians, and psychologists

*Professional historians*, most lacking expertise and without own psychological studies, have become increasingly prominent in the research field of the history of psychology in the US and in the German-speaking countries (however, their aspirations are more frequently the nonfiction book list rather than scientific publications). This follows the stronger and older trends in the domains of the history of the physical and natural sciences. Again, Danziger’s (1994, p. 467) critical remark about the “purely pedagogical role” (and—it should be added—the public and popular scientific role) of a “shallow history”, which is characteristic for the “natural rather than the human sciences”, is in line with this observation.

In addition to this more popular science and nonfiction book list orientation of historians, there is an increasing body of research on the history of psychology (and other sciences) by professional historians in the wider context of the “*science of sciences*” (see, e.g., Dobrov 1969; Krampen and Montada 2002; Ossowska and Ossowski 1964). This research stream attempts to cross-sectionally identify and analyze commonalities of all sciences and the humanities as well as the differences between them in the traditions of the philosophy of science, science politics, science psychology, science sociology, methodology, and the history of sciences, too. Characteristic is a research project oriented, problem-centered approach, which is limited in time and mission oriented (e.g., in the context of celebrations and academic ceremonies, but sometimes also with the explicit objective to obtain an external perspective on a—potential—critical, problematic, or negative history of an institution, theory, or something similar). Such external perspectives have the potential to be more objective and neutral and may provide new, perhaps invigorating reports and interpretations. However, the genuine psychological background in theories, constructs, terminology, methods, etc. is frequently missing in such pure historical, often rather statistical (Danziger 1995) analyses (sometimes enriched by various historical anecdotes). Therefore, psychology should not simply give the history of psychology away to another profession.

To sum up, it is obvious that interdisciplinary cooperation of specialist historians and psychologists is optimal, at least at times. In this case, both the experience of the collaborators and the rule of thumb that interdisciplinary cooperation on interdisciplinary subject matters and topics leads with higher probability to good or even excellent results (e.g., a psychologist and a sociologist join together to coauthor a book in the subdiscipline of social psychology). Even better would be transdisciplinarity in research on and teaching of the history of psychology—however, actual transdisciplinarity occurs in the mind of one individual (i.e., intraindividual), not between two or more individuals in the sense of inter- or multidisciplinarity (Stock 2012). Because such transdisciplinary minds are seldom, we have to aim for interdisciplinary cooperation, at least at times and for certain projects, but not universally, because this would possibly lead to the danger of giving the history of psychology away (and—perhaps—relieve psychology and psychologists from the “burden” of engaging in research on and the teaching of the history of psychology).

Results of scientometric analyses executed by psychologists are recently rarely and scattered randomly published with reference to the historiography of psychology in psychological journals and books: Focal points are research trends and deficits in selected countries (e.g. for Spain: Carpintero et al. 2010; Lafuente and Herrero 2003; for the German-speaking countries: Schui et al. 2014) and related international comparisons (e.g., Bornmann et al. 2012; Krampen and Montada 1998), the historiography of special topics of psychological research (e.g., Simonton 2014) and special epochs of psychology (see, e.g., Green et al. 2013, 2104, 2015), scientometric contributions to evaluative topics (e.g., Krampen 2008), whereas methodological issues of scientometrics are rarely brought into focus (e.g., Krampen 2008; Simonton 2006). Thus, this is a fruitful area for research cooperation of *expert scientometricians* and psychologists, which can contribute to the objective to give again “history of psychology (…) an impact” (Pettit and Davidson 2014, p. 709) within psychology and in its neighboring sciences.

### Research on and teaching of the history and the time long past of psychology

The last, but not the least, impulse and another of Danziger’s (1994) arguments: In his 1994 publication, he reflects on the “potential effects of critical historical studies on conceptions of the subject matter of psychology” (Danziger 1994, p. 467), and in 1997 he published the important book entitled “*Naming the mind: How psychology found its language*.” This problem history of psychological terminology includes the “long past” (Ebbinghaus 1908, p. 7) of psychology, that is, psychological conceptions and the understanding of the subject matter of psychology in its historical development and cultural contexts before psychology was established as a discrete discipline within the canon of sciences in the late nineteenth century. One of the internationally lesser-known German-language contributions (e.g., Jüttemann 2015; Jüttemann et al. 2005; Pongratz 1984) is introduced in the following in some detail.

Pongratz (1984) has provided a thorough description of the problem history of central concepts of (pre-) psychology going back in time for each concept as far as possible, and in some cases this extends partly back to the Egyptian and classic antique mythologies and religions. This is done for seven groups of conceptions:

1. Conceptions of *mind* (as a static concept) from the differentiated animism and Aristoteles’ 3-level model of mind over theonomous (i.e., doctrinaire theological) conceptions in the middle ages and rationalistic conceptions during the Enlightenment up to its increasing displacement by the modern conception of the person;
2. Conceptions of *psychic (inner) life* (as a dynamic concept) from Aristoteles’ concepts of the development of inner life by sensations and associations over more differentiated associative conceptions during the Enlightenment (e.g., David Hartley’s [1705–1757] theory of vibratiuncles), psychophysical and neuropsychological materialism in phrenology and organology up to dynamics of inner life in the “Vermögenspsychologie” (i.e., the differentiation of special human capacities or abilities, e.g., thinking, feeling, free will, etc.);
3. Conceptions of *consciousness* from its enduring roots in classic antique Roman Stoa and philosophy being defined by intentionality and reflexivity over the more differentiated conceptions in Scholastics, Husserl’s phenomenology, Brentano’s act psychology, Wundt’s introspective experimental psychology up to recent psychological theories of action;
4. Conceptions of *unconsciousness* from its experiential roots in mythologies and occultism over conceptions in depth psychology, psychoanalytic and neopsychoanalytic theories as far as volitional-motivational conceptions (e.g., irrational emotions and defense mechanisms) and perceptual-cognitive conceptions (i.e., subliminal perception and incidental learning) in modern psychology;
5. Conceptions of *behavior* from its scientific analyses in reflexology over the different variations in behaviorism and neo-behaviorism as up to its treatment in modern action theories as automatisms (vs. autonomisms);
6. Conceptions of *experience* with its actual-genetic features of temporality, holism, and immediacy in Gestalt psychology up to introspection and the principle of empathic understanding in phenomenology, hermeneutics, and humanistic psychology and psychotherapy;
7. Conceptions of *cognition and action* from Narciss Ach’s analyses of volitions and acts over Tolman’s cognitive maps, J. B. Rotter’s social learning theory of personality, and the TOTE-unit from Miller, Galanter, and Pribram as far as recent cognitive psychology and action theories;
8. For the last two or three decades, as number (8), historical and modern *conceptions of the brain* in neuropsychology and interdisciplinary neurosciences may be added (Pongratz’ book was published in 1984).

Thus, the argumentation is confirmed that it is worth exploring, investigating, and teaching the problem history of psychology and the understanding of the subject matter of psychology and its historical development in cultural contexts. This not only beneficial for promoting a better understanding of the development of mankind and cultural anthropology, in which concepts of mind and psychological conceptions were and are very significant, but for personal, social, and sociopolitical insights as well. The problem history of psychological conceptions contributes not only to the understanding of the common ground of the three large monotheistic religions (to which Buddhism may be added without second thoughts), but also to a better understanding of the development of the sciences and humanities in the classic antique, the classic era of Enlightenment, and in the recent age of communication, which may be designated as a “late stage of the so-called Enlightenment” (Zeh 2014, p. 250; translation provided by the author). This stage argues critically against authorities as well and is characterized by technological revolutions of the Internet and digitalization in strong combination with a revolution of human awareness. This was, at any time, and remains in our modern times a significant educational objective.

## Conclusions

Based on scientometric analyses of the PsycINFO and PSYNDEX databases, the present findings provide historiographical confirmation that research on and teaching of the history of psychology have sustained a loss and decline in recent psychology and—therefore—may have a limited future. Publications with a main focus on these topics decreased markedly since the 1990s in the German-speaking and Anglo-American research communities. Because this is also true for publications on the history of psychology intended for use in teaching, there are also no serious hints for a more “pedagogical role” of a “shallow history” (Danziger 1994, p. 467) in the field.

History of psychology seems to vanish from undergraduate and graduate psychology curricula and historical research appears to have been placed in a niche. With reference to the absolute and relative frequencies of psychological publications, the status of the history of psychology has become marginal in general and—even worse—in the different subdisciplines of psychology. There is only one exception: A very large number of publications documented in PsycINFO and PSYNDEX explore the history of psychoanalytic and neo-psychoanalytic theory and psychotherapy. A closer look at this literature reveals that it not only addresses the “great men” and their scientific contributions, but primarily historical contributions concerning the epidemiology and etiology of mental disorders in different epochs and different cultural contexts as well as the significance of developmental contexts for personality development. Thus, in psychoanalysis and depth psychology, the history of psychology is thriving and very productive, whereas nearly all other subdisciplines of psychology ignore their history to a large extent—at least as a focus in research and publications.

Even worse than this lack of regard is the fact that the small share of publications on the history of psychological subdisciplines is difficult to search for, because—particularly in PsycINFO—these publications are seldom correctly documented with the classification code (CC) of the subdiscipline. In reality these are frequently assigned no CC at all. This is a serious limitation of PsycINFO, which results from the more automatic and computerized and thus inherently less differentiated documentation procedure (while the documentation in PSYNDEX accords with the academic library principle of autopsy performed by specialist psychologists). This shortcoming, however, does not affect the present scientometric results on the history of psychology because they are based primarily on searches of the subject heading (SH) and secondly on searches of the classifications provided by the database following the CC schema delineated in the *Thesaurus of Psychological Index Terms* (Gallagher Tuleya 2007; ZPID 2011).

Furthermore, the scientometric results show that, since the 1970s, the published literature on the history of psychology is losing its character as a “book science” and is becoming a “journal science”. This follows the contemporary trend in psychology at large to publish short, but many journal articles (as well as some book chapters), and although this accords with the dissemination model of the natural sciences, it does not with that of the human sciences and the humanities (Danziger 1994). However, one of the future opportunities for research on the history of psychology to optimize its visibility may be provided by increasing the number of publications not only in specialized journals on the history of psychology, but also in journals and conference proceedings of the other subdisciplines of psychology. Of course, this must be accompanied by more intensive historical analyses in authored and—perhaps—edited books, which have the potential to draw interest of psychologists working in research in other subdisciplines and in applied settings. Up-to-date hot topics may refer to, for example, historical analyses of the boom in neuropsychology and neurosciences, human information behavior and the evaluation of the reliability of information and references, especially in the epochs with dramatically changing media (e.g., invention of printing in the fourteenth century and recent digitalization and Internet), and the history of the interdisciplinary cooperation of psychology with psychiatry, medical science, criminology, educational sciences, etc. Students’ and colleagues’ historical interests can be best awakened if the historical question and analyses under study match the current or anticipated field of work or—perhaps also—the *Zeitgeist* or sociopolitical occurrences and developments.

Another impulse for promoting up-to-date research on and the teaching of the history of psychology refers to opening up and using even more the advantages of digitalization and the Internet. Specifically, as complements to the well-tried historical methods, other and new methods are required in research on and teaching of the history of psychology for the reduction and handling of information overload and “big data.” This could include, for example, scientometrics, time series analyses, semantic technologies, visual displays of information, etc. (see above), and they all have the advantage to be motivating for students and colleagues. At once, these (mostly quantitative) methods may open the gate to allow further steps on the way to a conceptual and methodological integration of qualitative and quantitative approaches in the natural and the human sciences.

Internet and digitalization are not ahistorical. Quite the contrary, these technologies enlarge and provide storage for information in a huge, disproportional manner, and the (open access) user must be supported by experts in his or her efforts to search, structure, restructure, integrate, and evaluate information. Information without experts and without guards and guardians as well (Keen 2015) is often not fully correct, sometimes it is tainted, and sometimes both. Living and working in a “late stage of the so-called Enlightenment” (Zeh 2014, p. 250; translation provided by the author), which is characterized by technological revolutions of the Internet and digitalization that is strongly linked with a revolution of human awareness, requires such experts and specialists for the history of psychology and scientometrics, who should belong to these experts.

In closing I would like to make a few brief remarks on Danziger’s (1994, p. 467) hopes for “developments which provide a more favourable context for critical historical scholarship”. One of his three hopes refers to “the international diversification of psychology” (p. 467). Indeed, there are some indications in the form of small hints that US-American psychologists are beginning to reflect upon the international dominance of US psychology. For example, Arnett (2008, 2009) started a debate on the “the neglected 95 %” and the question “why American psychology needs to become less American” (Arnett 2008) with the preliminary result and insight that this poses “a challenge to psychology’s philosophy of science” (Arnett 2009). However, this is not at all reflected in PsycINFO, because it is clearly dominated by Anglo-American and English-language publications: Approximately 65 % of the documents are from the Anglo-American countries, approximately 90 % of the documents refer to English-language publications, i.e., approximately 25 % are from the rest of the world; less than 2 % of the documents are English and German publications from the German-speaking countries. These numbers depict a rather low quota for a database that is internationally renowned. Yet they converge with recent self-criticism of US-American psychology to neglect or overlook, to a great extent, the samples, psychological research, and publications in “the rest of the world”. The scientometric results presented here confirm this situation: Only 512 of the 8130 English- and German-language publications on the history of psychology from the DACHLL countries documented in PSYNDEX are documented in PsycINFO as well. Thus, the coverage (i.e., 6.3 %) is very low and speaks neither for the envisioned international diversification of psychology in general nor of research on and teaching of the history of psychology.
